# Salvage Therapy for Refractory Aids-Related Primary Central Nervous System Lymphoma 

**DOI:** 10.1155/2012/343491

**Published:** 2012-09-18

**Authors:** Hugo Ferro, Eduardo Parino

**Affiliations:** Department of Internal Medicine, Clínica y Maternidad Suizo, Argentina, Avenida Pueyrredon 1461, C1118AAE Buenos Aires, Argentina

## Abstract

A 27-year-old male patient presented with speech disorders and multiple brain masses on MRI evaluation. He tested positive for HIV. A sterotactic biopsy diagnosed primary central nervous system lymphoma (diffuse large B-cell lymphoma). After two cycles of high-dose metotrexate (HD-MTX-)-based chemotherapy, the tumor progressed. He underwent whole brain radiotherapy achieving complete response. Six cycles of consolidating immunochemotherapy with rituximab-temozolomide were administered after radiation. Forty-three months after remission, he has not recurred and his neurological status is optimal. Younger HIV patients with refractory PCNSL and preserved immune function can face salvage therapy successfully achieving long term remissions with no remarkable neurotoxicity.

## 1. Introduction

Primary central nervous system lymphoma (PCNSL) arises in and is generally confined to central nervous system. It accounts for 15% of all non-Hodgkin lymphoma seen in AIDS patients. Although the prognosis of HIV-associated PCNSL has improved after the introduction of HAART, it continues being an aggressive and challenging disease that demands very often unrewarding efforts. About 10–35% of patients do not respond to initial therapy, and more than half of responders will relapse during the followup [1].

Herein, we report an HIV patient with primary brain B-cell lymphoma who progressed under initial chemotherapy and achieved long lasting complete remission after salvage therapy with radiation and combined immunochemotherapy.

## 2. Case Report

**Figure 1:**
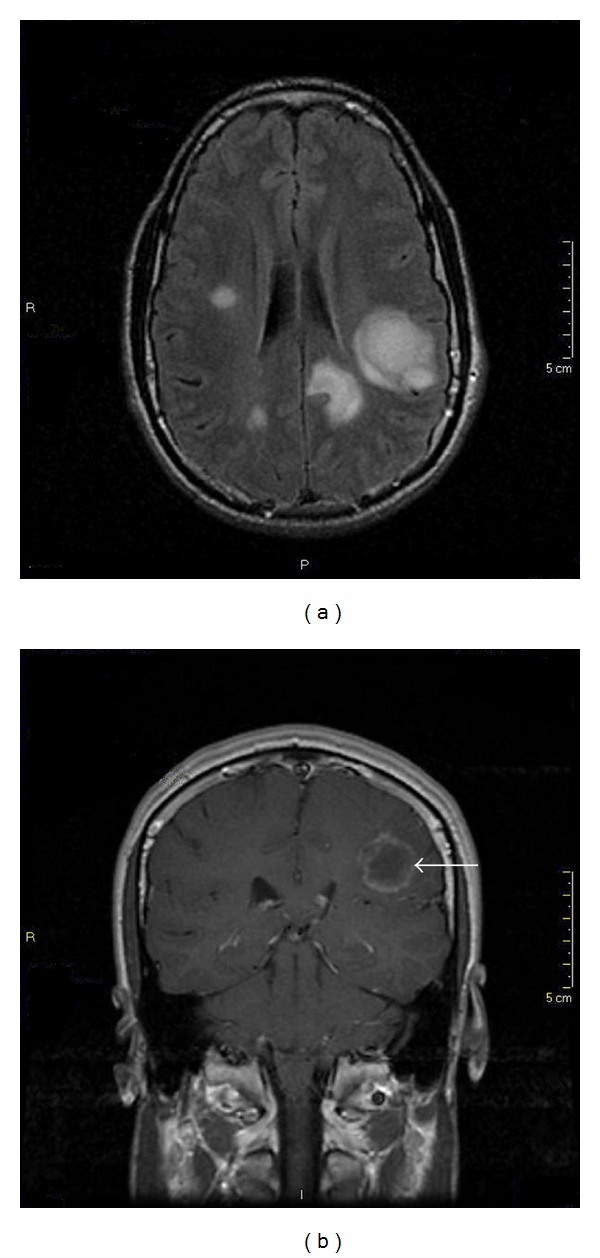
Initial MRI scan of the brain. An axial FLAIR image shows multiple hyperintense bilateral brain masses involving mainly left parietal lobe and splenium of corpus callosum (a). A coronal T1-weighted image (b) obtained after contrast administration shows gadolinium rim-enhancement of the main lesion (arrow).

A 27-year-old male patient who had recently tested positive for HIV was admitted at our facility with language disturbances in late May 2008. Physical exam and laboratory tests were unremarkable. A magnetic resonance imaging (MRI) scan documented multiple bilateral brain masses hypointense to gray matter on the T1-weighted images and hyperintense on the T2-weighted and FLAIR sequences. The two main lesions on left parietal lobe and splenium of corpus callosum showed enhancement after the administration of gadolinium (Figure 1). Treatment for toxoplasmosis with pyrimethamine-sulfadiazine was started. Ten days later the patient developed persistent high fever, maculopapular skin rash, multiple lymphadenopathies, and facial edema. Cervical lymph-node biopsy was nondiagnostic. Chest and abdominal CT scan were performed with no findings. It was interpreted as delayed-type hypersensitivity to sulfas and one single dose of methylprednisolone was administered. The patient became afebrile and symptoms resolved in 48 hours. Toxoplasmosis treatment was discontinued. Serology for Toxoplasma IgG was negative, CD4+ cells count: 149/mm^3^ (15%) and HIV viral load: 2550 RNA copies/mL (log⁡⁡3.4). HAART was started with tenofovir emtricitabine plus efavirenz. A new brain MRI did not reveal any changes. Spectroscopy MR of the left parietal mass revealed a decreased N-acetylaspartate peak, an increased creatine/choline ratio and a lipid/lactate peak.

**Figure 2:**
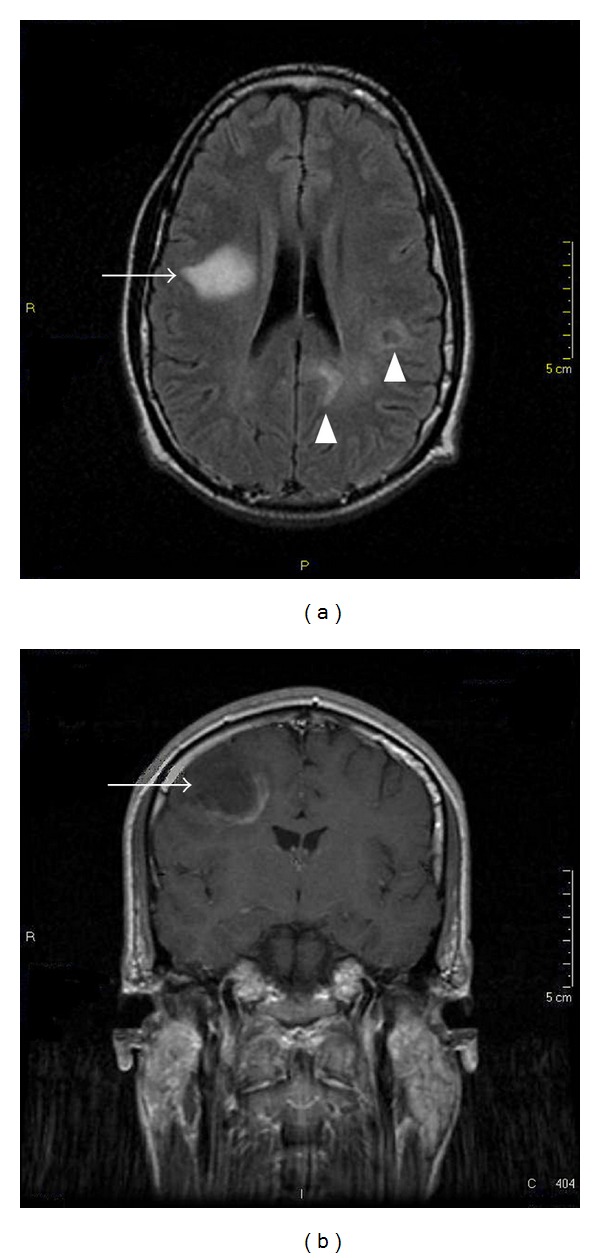
MRI scan after initial therapy. After two cycles of HD-MTX-based chemotherapy, the axial FLAIR image (a) shows good response of left brain masses (arrowheads) but progression of right frontal one (arrow) with contrast reinforcement on the T1-weigthed sequence with gadolinium (b).

**Figure 3:**
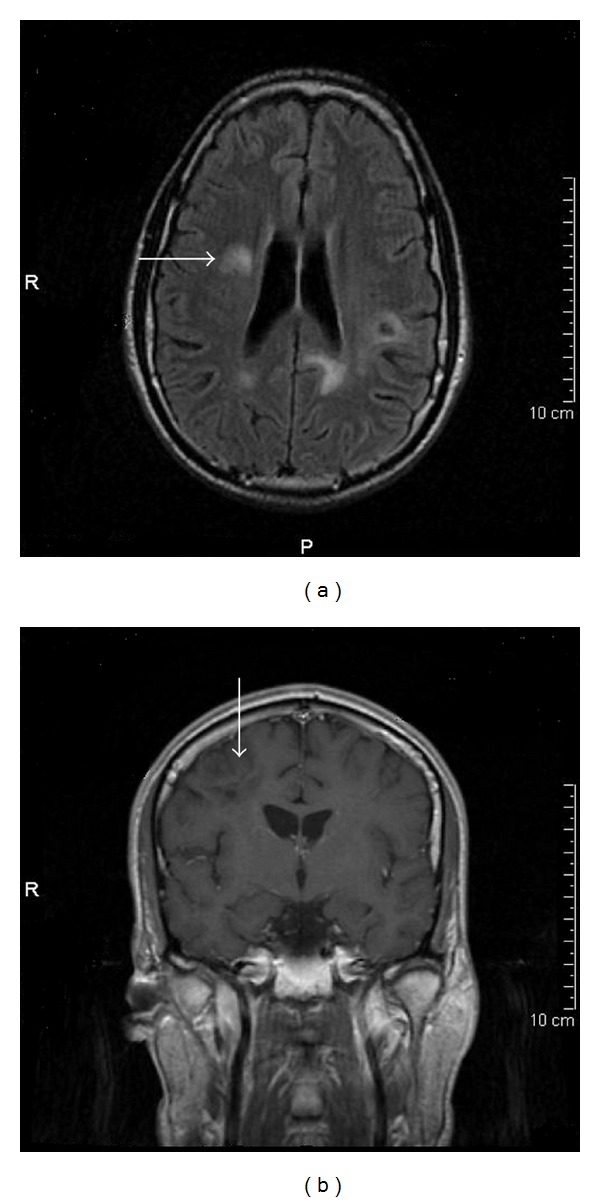
MRI scan after WBRT. After radiation therapy axial FLAIR image shows marked-size reduction of right-frontal mass (a) with no contrast enhancement in T1-weighted image after administration of gadolinium (b). According to International Revised Criteria for PCNSL Response Assessment, it was considered a complete response. Subsequent follow-up scans did not display any changes on remaining lesions and a FDG-PET scan undergone after completion of immunochemotherapy confirmed no residual tumoral activity.

 Two weeks later a stereotactic biopsy was performed. The histopathological examination diagnosed diffuse large B-cell lymphoma. There was no evidence of extension of the disease beyond the central nervous system. Bone marrow biopsy was unrevealing. In mid July, he started receiving HD-MTX-based chemotherapy. By then, after 4 weeks of HAART, CD4+ cell count had risen to 439 (31%). Karnofsky Performance Status (KPS) score was 90. He was delivered a modified version of MATILDE schedule [2]: methotrexate 3,5 grs/m^2^ on day 1, cytarabine 1,7 grs/m^2^ × 2 day on day 2, idarrubicin 13 mg/m^2^ on day 2, and cyclophosphamide 900 mg on day 3 recycling every 3 weeks. After two cycles, a brain MRI showed shrinkage of left hemisphere lesions but significant enlargement of the right frontal one with contrast-enhancement (Figure 2). The patient developed partial left facial palsy and clumsy left hand. He was started on steroids (dexamethasone 8 mg tid orally) and whole brain radiation therapy (WBRT) was indicated. He initially received 40 Gy and then a 10 Gy boost. Corticosteroids were stopped two weeks after initiating radiation. By mid October, a new MRI revealed no contrast-enhancement lesions (Figure 3). 

 Then, six cycles of rituximab-temozolomide (RTX-TMZ) were administered: RTX 375 mg/m^2^ intravenously on day 1 and TMZ 150 mg/m^2^ from day 1 to 5 every 28 days. From second cycle on the dosage of TMZ was reduced to 100 mg/m^2^ because of grade 3 neutropenia. During consolidating therapy residual nonenhancing MRI lesions did not modify. They were assumed as radionecrosis and this impression was confirmed two months later when a FDG-PET scan was performed without displaying any uptake. By this time, May 2009, CD4+ count had dropped to 249 cells/mm^3^ (18%) but viral load remained undetectable.

 In October, a neurocognitive study only showed subtle memory deficits. His performance status was excellent. Since then he has been controlled every three months, the first two years and then every six months. Forty-three-months after achieving remission, there is no evidence of relapse or substantial treatment-related neurotoxicity. CD4+ cell count has recovered to 427/mm^3^ (24%).

## 3. Discussion

High-dose methotrexate (HD-MTX-)-based regimens have become the initial approach for treating patients with newly diagnosed primary CNS lymphomas, with response rates of 70–80% reported [2, 3]. Non responders exhibit a poorer prognosis. Reni et al reported than median time to death was 14 months for patients who underwent salvage therapy [1]. Similar results were obtained by Gavrilovic and colleagues [4]. Moreover, the optimal treatment for those who progress has not been determined yet. Traditionally whole brain radiation therapy (WBRT) was suggested as the treatment of choice for refractory disease after initial chemotherapy. Response rates are high—in fact more than 70% has been reported between complete (CR) and partial responses (PR) —with a median survival rate ranging from 11 to 16 months [5, 6]. Age less than 60 years and achievement of a CR seem to be predictors of better survival. 

 There is some evidence that relapses that occur after combined chemotherapy and WBRT are associated with an unfavorable prognosis [7]. In the case we report, in order to consolidate the response obtained after WBRT and delay the recurrence, six cycles of combination therapy of RTX plus TMZ were indicated. Several reports have established the potential of this combination as induction, consolidation, and maintenance therapy for PCNSL with modest toxicity [8–11]. TMZ is an oral alkylating agent with good CNS penetrance and well tolerated. Its main toxicity, myelosuppression, is usually dose-related and not severe at the common schedule of 150 mg/m^2^/day. Rituximab, a chimeric monoclonal antibody against CD20 antigen, has an elevated molecular weight and achieves only a CSF concentration of 0.1% of that in the serum after intravenous administration. Nevertheless, there is evidence that suggests that rituximab would increase cytotoxic effects of alkylating agents by inhibiting interleukin-10 that results in downregulation of Bcl-2 and sensitization of CD20+ B cells lymphoma to apoptosis [12]. Therefore, the sequence of administration, RTX followed by TMZ is emphasized, because of the synergy described. 

 It is difficult to determine the real contribution of combined immunochemotherapy to prolong the response achieved after WBRT; this subject would deserve further investigation. We cannot underestimate either the role of HAART and its contribution to improve immune status and prevent relapse [13, 14]. 

 Most published trials and anecdotal reports on this devastating and rare disease refer to immunocompetent hosts. It is evident, however, that HIV patients are also able to undergo aggressive treatments with good tolerance and scarce morbidity. Indeed, several studies have endorsed the use of anti CD20 with a preserved CD4+ cell count at the start of therapy without a significant increase in the rate of fatal infections [15]. In any case, even in this setting of rapid progression of the disease and lack of response to initial chemotherapy, our experience demonstrates that a sustained remission can be obtained without significant late neurotoxicity, especially in younger patients with good performance status.

## References

[1] Reni M, Ferreri AJM, Villa E (1999). Second-line treatment for primary central nervous system lymphoma. *British Journal of Cancer*.

[2] Ferreri AJM, Dell’Oro S, Foppoli M (2006). MATILDE regimen followed by radiotherapy is an active strategy against primary CNS lymphomas. *Neurology*.

[3] Poortmans PMP, Kluin-Nelemans HC, Haaxma-Reiche H (2003). High-dose methotrexate-based chemotherapy followed by consolidating radiotherapy in non-AIDS-related primary central nervous system lymphoma: European Organization for Research and Treatment of Cancer Lymphoma Group Phase II Trial 20962. *Journal of Clinical Oncology*.

[4] Gavrilovic IT, Hormigo A, Yahalom J, DeAngelis LM, Abrey LE (2006). Long-term follow-up of high-dose methotrexate-based therapy with and without whole brain irradiation for newly diagnosed primary CNS lymphoma. *Journal of Clinical Oncology*.

[5] Nguyen PL, Chakravarti A, Finkelstein DM, Hochberg FH, Batchelor TT, Loeffler JS (2005). Results of whole-brain radiation as salvage of methotrexate failure for immunocompetent patients with primary CNS lymphoma. *Journal of Clinical Oncology*.

[6] Hottinger AF, Deangelis LM, Yahalom J, Abrey LE (2007). Salvage whole brain radiotherapy for recurrent or refractory primary CNS lymphoma. *Neurology*.

[7] Omuro AM, Abrey LE (2006). Chemotherapy for primary central nervous system lymphoma. *Neurosurgical Focus*.

[8] Enting RH, Demopoulos A, DeAngelis LM, Abrey LE (2004). Salvage therapy for primary CNS lymphoma with a combination of rituximab and temozolomide. *Neurology*.

[9] Wong ET, Tishler R, Barron L, Wu JK (2004). Immunochemotherapy with rituximab and temozolomide for central nervous system lymphomas. *Cancer*.

[10] Wong ET, Barron L, Bloom J, Wu J (2004). Durable responses from immunochemotherapy with rituximab and temozolomide in patients with CNS lymphomas. *Cancer Biother Radiopharm*.

[11] Santisteban M, Nieto Y, De la Cruz S, Aristu J, Zubieta JL, Fernández Hidalgo O (2007). Primary central nervous system lymphoma treated with rituximab plus temozolomide in a second line schedule. *Clinical and Translational Oncology*.

[12] Alas S, Bonavida B (2001). Rituximab inactivates signal transducer and activation of transcription 3 (STAT3) activity in B-non-Hodgkin’s lymphoma through inhibition of the interleukin 10 autocrine/paracrine loop and results in down-regulation of Bcl-2 and sensitization to cytotoxic drugs. *Cancer Research*.

[13] Hoffmann C, Tabrizian S, Wolf E (2001). Survival of AIDS patients with primary central nervous system lymphoma is dramatically improved by HAART-induced immune recovery. *AIDS*.

[14] Skiest DJ, Crosby C (2003). Survival is prolonged by highly active antiretroviral therapy in AIDS patients with primary central nervous system lymphoma. *AIDS*.

[15] Wyen C, Jensen B, Hentrich M (2012). Treatment of AIDS-related lymphomas: rituximab is beneficial even in severely immunosuppressed patients. *AIDS*.

